# Case Report: Pulmonary and gastrointestinal involvement in a rare case of Waldenström’s Macroglobulinemia in French Guiana

**DOI:** 10.3389/fmed.2025.1551600

**Published:** 2025-05-07

**Authors:** Houari Aissaoui, Dana Gaudard, Ghazi Hadj Amara, Alolia Aboikoni, Yanna Lingibe, Dominique Louvel

**Affiliations:** ^1^Department of Medicine B, Cayenne Hospital Center - Andrée Rosemon, Cayenne, France; ^2^Department of Internal Medicine, Cayenne Hospital Centre, Cayenne, France; ^3^Department of Pathology, Cayenne Hospital Centre, Cayenne, France

**Keywords:** Waldenström’s Macroglobulinemia, pulmonary and digestive endoscopy, extra-hematopoietic involvement, lymphoplasmacytic infiltration, systematic biopsies

## Abstract

**Background:**

Waldenström’s Macroglobulinemia (WM) is a rare lymphoproliferative disorder characterized by the proliferation of lymphoplasmacytic cells producing monoclonal immunoglobulin M (IgM). Extra-hematopoietic involvement, although rare, can affect the gastrointestinal tract in approximately 4% of cases and the lungs in 3–5% of cases. This case highlights the diversity of extramedullary manifestations in WM and underscores the importance of performing pulmonary and gastrointestinal endoscopies with systematic biopsies for optimal disease management.

**Case presentation:**

We report the case of a 60-year-old Haitian man diagnosed with WM in January 2023 following an episode of acute respiratory failure. Biopsies revealed lymphoplasmacytic infiltration in both the bronchial and gastrointestinal mucosa. Biopsies revealed lymphoplasmacytic infiltration in both the bronchial and gastrointestinal mucosa, despite normal macroscopic findings during gastrointestinal endoscopy.

**Conclusion:**

This case emphasizes the importance of systematic biopsies during endoscopic examinations, even in the absence of visible lesions, to detect lymphoplasmacytic infiltration in WM. These findings could help in changing the approach to disease management and improving patient outcomes.

## Introduction

Waldenström’s Macroglobulinemia (WM) is a rare form of non-Hodgkin lymphoma characterized by the excessive production of monoclonal immunoglobulin M (IgM) due to lymphoplasmacytic tumor infiltration (1). First described by Waldenström in 1944 in two male patients (2, 3). The annual incidence in France is 1,300 cases with male predominance (sex ratio: 2.1) and the median age of diagnosis is 73 years (4). The clinical and hematologic manifestations of WM are well-documented, reflecting both the monoclonal activity of IgM and tissue tumor infiltration (5, 6). Bone marrow infiltration occurs in nearly 100% of cases, as reported by Kapoor et al. while extra bone marrow infiltrations are less common, with gastrointestinal involvement in about 4% of cases (7), pulmonary involvement in 3%–5% (1). The endobronchial involvement is particularly rare.

Our patient presents a distinct case of Waldenström’s Macroglobulinemia with pulmonary parenchymal involvement, lymphoplasmacytic infiltration of the bronchial mucosa with a distinctive petechial endoscopic appearance, and lymphoplasmacytic infiltration of the digestive mucosa, despite a normal gastrointestinal endoscopic appearance. In French Guiana, lymphoproliferative diseases are rarely reported, making this case valuable addition to the local medical literature.

## Case report

We report the case of a 60-year-old Haitian man diagnosed with Waldenström’s Macroglobulinemia in January 2023, following an episode of acute respiratory failure requiring a 10-days stay in the intensive care unit. The patient was treated for secondary bacterial infection following H1N1 influenza viral pneumonia, and a hyper viscosity syndrome, which improved gradually with plasma exchange therapy.

The patient’s bone marrow aspirate revealed a polymorphic lymphoid infiltration, including lymphocytes (58%), lymphoplasmacytic cells and plasma cells (10%). Serum protein electrophoresis and immunofixation demonstrated a monoclonal immunoglobulin M of both kappa and lambda types, with a peak concentration of 45.8 g/L. The MYD88 mutation was not detected.

Thoraco-abdominal-pelvic CT imaging showed multiple areas of parenchymal consolidation with ground-glass opacities (involving 50% of the left lung and 70% of the right lung). Following a multidisciplinary team (MDT) discussion, the patient received six cycles of chemotherapy with Cyclophosphamide and Rituximab from February 15, 2023 to July 18, 2023 in the onco-hematology department. At the end of the treatment, the patient discontinued follow-up.

In October 2023, the patient experienced a clinical recurrence, presenting with dyspnea, hemoptysis, purulent sputum, and general decline. He was admitted to the pulmonary unit, where vital signs indicated an oxygen desaturation of 92% and fever of 38.4°C. Laboratory findings showed an inflammatory syndrome with leukocytosis of 14,000 G/L, C-Reactive Protein (CRP) of 380 mg/L, microcytic anemia with hemoglobin at 8.8 g/dL, iron deficiency with transferrin saturation at 2%, serum iron at 7.5 μmol/L, Ferritin at 20 ng/mL, a platelets at 190 G/L, prothrombin time (PT) at 81%, and a total protein level of 111 g/L. Hepatic function was normal, and vitamins B9 and B12 were within normal limits.

**FIGURE 1:**
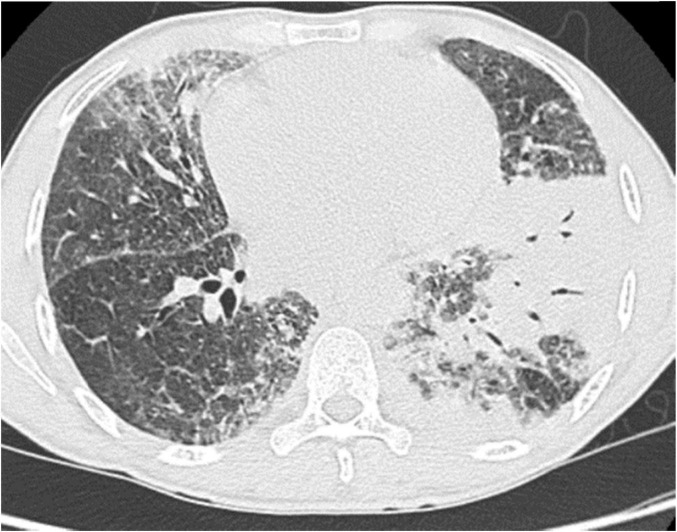
Chest CT scan showing chronic pulmonary consolidation in the left lower lobe.

Chest scan revealed parenchymal consolidations in both lung fields, predominantly in the left lung (Figure 1), with mediastinal and sub-diaphragmatic infiltration, and minimal right-sided pleural effusion.

Initial empiric antibiotic therapy with Rovamycine and Cefotaxime was administrated for 7 days, resulting in clinical and biological improvement, with CRP decreasing to 9 mg/dL.

**FIGURE 2:**
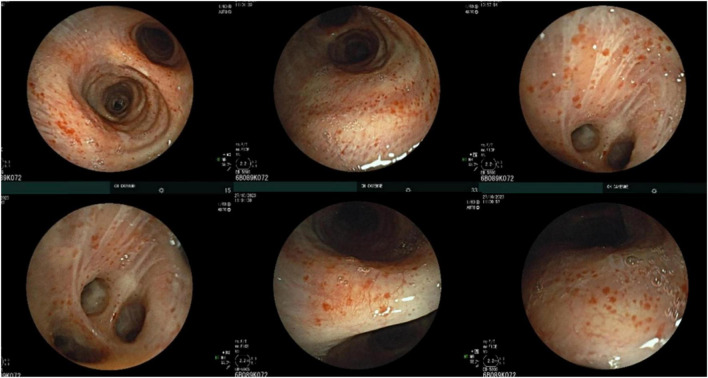
Bronchoscopic appearance with diffuse petechial involvement of the tracheobronchial mucosa.

Bronchoscopy revealed a diffuse petechial appearance of the tracheobronchial mucosa (Figure 2). Bronchoalveolar lavage (BAL) fluid analysis showed a cellularity of 450,000 cells/mL, comprising 38% macrophages, 56% lymphocytes, 6% neutrophils, and rare eosinophils. Predominant plasma cells were observed, some of which were atypical and expressed CD45 and CD20, consistent with bronchoalveolar involvement by Waldenström’s Macroglobulinemia.

Staged bronchial biopsies of the spurs revealed massive infiltration by a monomorphic B-cell proliferation (CD20, CD79), with some plasma cells (CD138, IgM) infiltrating mucous-secreting bronchial acini (Figure 4).

**FIGURE 3:**
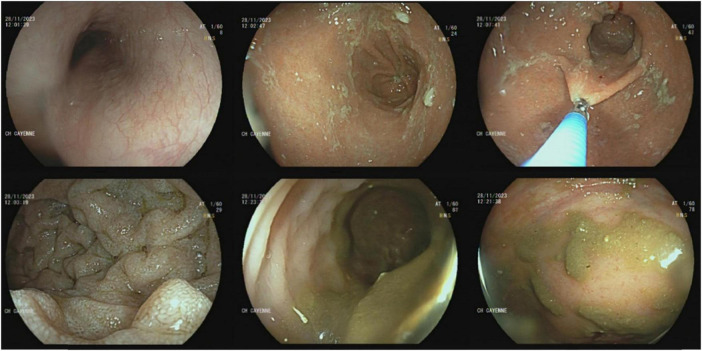
Gastrointestinal endoscopy and rectoscopy visualizing the absence of macroscopic lesion of the digestive mucosa.

**FIGURE 4:**
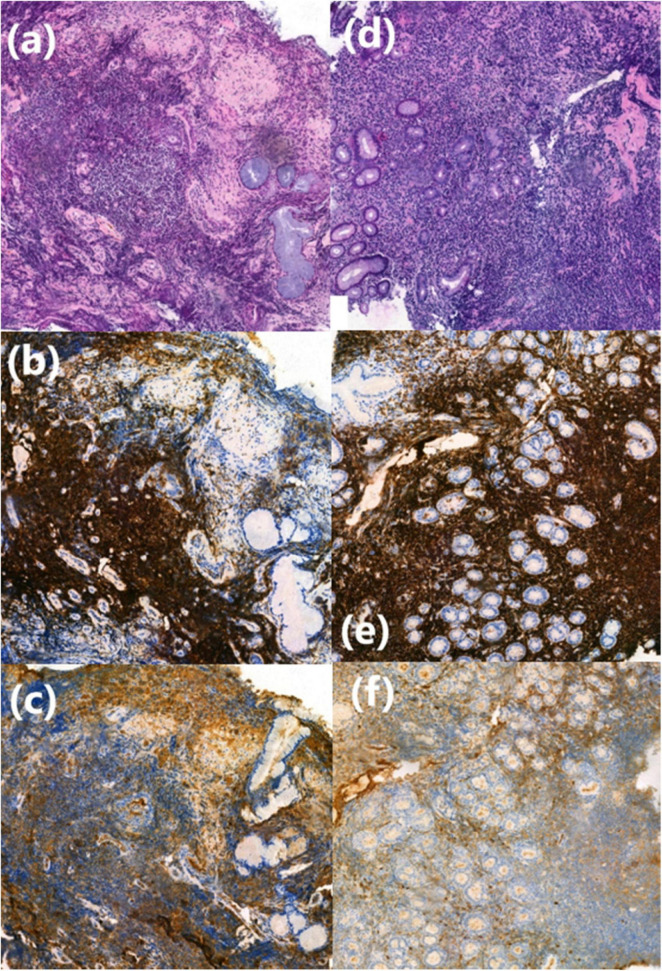
Histological finding from bronchial and digestive biopsy specimens. **(a)** Lymphoplasmacytic cell infiltration in the bronchial chorion, HES (Haematein-Eosin-Saffron), (×10). **(b,c)** CD20+ and IgM+ lymphoplasmacytic cells shown by immunohistochemistry (×20). **(d)** Lymphoplasmacytic cell infiltration in the fundic chorion, HES (Haematein-Eosin-Saffron), (×10). **(e,f)** CD20+ and IgM+ lymphoplasmacytic cells shown by immunohistochemistry (×20).

To investigate the iron-deficiency anemia, an upper gastrointestinal endoscopy and rectoscopy were performed. No macroscopic lesions were observed (Figure 3). However, histological analysis of digestive biopsies revealed diffuse lymphoplasmacytic infiltration in the gastric antrum and rectal mucosa (Figure 4).

The patient was subsequently referred to the onco-hematology department for a new MDT discussion, a second line treatment with Ibrutinib (420 mg/day) and plasmapheresis was initiated on January 2024. Unfortunately, the patient passed away in October 2024.

**Table showing the timeline of cares:**

**Table:** 

Date	Event	Outcome
Jan 2023	Diagnosis of WM following acute respiratory failure	– Lymphoplasmacytic infiltration in bone marrow + monoclonal IgM.-Chest scan: Parenchymal consolidations.
Feb 2023	MDT discussion	– 6 cycles of Cyclophosphamide and Rituximab.
**Discontinuation of medical follow-up**		
Oct 2023	Clinical recurrence with respiratory distress.	– Clinical and biological improvement under an antibiotic therapy. -Chest scan: Persistent parenchymal consolidations.-Bronchoscopy: petechial appearance of tracheobronchial mucosa.-Lymphoplasmacytic infiltration of the bronchial mucosa-Lymphoplasmacytic infiltration in gastric antrum and rectal mucosa.
Jan 2024	New MDT discussion	– Switch to Ibrutinib 420 mg/day with plasmapheresis.
Oct 2024	**Patient’s death**	

## Discussion

Extramedullary infiltrations in Waldenström’s Macroglobulinemia (WM) are variable and rarely predominant (8). Rabiner et al. documented a case of WM without bone marrow infiltration, where pulmonary involvement was the sole manifestation, with dense lymphoproliferative infiltration of the bronchial mucosa evident on biopsy (9). The first case of pulmonary involvement in WM was described by Noah in 1956 (10), in a patient with widespread disease including bone marrow involvement. Since then, multiples case reports and reviews have documented a range of non-specific pulmonary manifestations, including parenchymal masses, reticular and nodular infiltrates, pleural effusions, and chylothorax (1, 11, 12).

In WM, the onset of respiratory symptoms warrants investigation for possible pulmonary involvement or secondary infection (13). In our case, despite recurring respiratory infections, chest CT findings were consistent with consolidations described in the literature.

As a non-Hodgkin lymphoma (NHL), endobronchial airway involvement was described by Rose et al. in 1986 (14). The authors reported two patterns observed in three cases of NHL and 17 additional cases reviewed from the literature: diffuse submucosal infiltrates (type 1) and a solitary mass in the central airways (type 2). Notably, Waldenström’s macroglobulinemia was not among the histologic patterns observed in these cases.

A literature review yielded three confirmed cases of WM with airway involvement observed during bronchoscopy, reported between 1992 to 2016. Sakai et al. documented multiple submucosal nodules in the trachea with significant lymphoplasmacytic infiltration on biopsy (15). Fadil and Taylor reported subtotal occlusion of the left upper lobe with diffuse mucosal irregularity (2). Hana et al. described an obstructive lesion in the right lower lobe with granulations in the trachea, necessitating interventional pulmonology procedures (16).

The petechial appearance of the trachea and bronchi in our patient’s bronchoscopy likely reflects mucosal bleeding, which can be a feature of WM. This bleeding has been attributed by some authors (17) to platelet dysfunction or an acquired Von Willebrand factor syndrome, both associated with the blood hyperviscosity seen in approximately 30% of WM cases (17). Additionally, mucosal bleeding may result from thrombocytopenia due to the tumor bone marrow infiltration.

Malabsorption syndrome, diarrhea, and gastrointestinal bleeding are frequently indicative of gastrointestinal tract involvement in WM (1). Our patient presented with iron-deficiency anemia and normal endoscopic findings, resulting from malabsorption secondary to lymphoplasmacytic infiltration of the digestive mucosa.

We are facing an aggressive form of WM with extra-medullary manifestations, particularly bronchial and gastrointestinal lymphoplasmacytic infiltrations, that progressed despite two lines of treatment, ultimately leading to the patient’s death. Recent research has highlighted the role of the immune microenvironment in WM pathogenesis, especially the interaction between WM cells and regulatory T cells via the CD40/CD40-ligand axis (18). This molecular understanding may lead to new therapeutic approaches.

## Conclusion

In this case, the simultaneous bronchial and gastrointestinal lymphoplasmacytic infiltrations represent an unusual manifestation of Waldenström’s Macroglobulinemia. Although pulmonary infiltration has been documented in several cases, concurrent endobronchial and gastrointestinal involvement is rarely reported.

This case highlights the importance of both bronchial and digestive endoscopic examinations in managing Waldenström’s Macroglobulinemia. Systematic biopsies, even in the absence of visible lesions, may reveal lymphoplasmacytic tumor infiltrations.

## Data Availability

The original contributions presented in this study are included in this article/supplementary material, further inquiries can be directed to the corresponding author.
